# Green innovation efficiency and multiple paths of urban sustainable development in China: multi-configuration analysis based on urban innovation ecosystem

**DOI:** 10.1038/s41598-023-40084-x

**Published:** 2023-08-10

**Authors:** Jinguang Guo, Yu Fu, Xuefu Sun

**Affiliations:** 1 School of Public Administration, Dongbei University of Finance and Economics, No. 217, Jianshan Street, Shahekou District, Dalian, 116025 Liaoning China; 2 School of Public Finance and Administration, Harbin University of Commerce, No.1, Xuehai Street, Songbei District, Harbin, 150000 Heilongjiang China

**Keywords:** Energy and society, Environmental economics, Sustainability

## Abstract

Enhancing the effectiveness of urban green innovation is a powerful strategy for advancing urban sustainability. A strong urban innovation ecosystem is a crucial building block for advancing urban green innovation’s effectiveness. We use the fsQCA method to investigate the pathways and models of innovation ecosystems to promote the green innovation efficiency of cities from a histological perspective, using 71 innovative cities in China as cases. This method is based on the DEA-SBM model to measure the green innovation efficiency of cities and the Necessary Conditions Analysis. According to our analysis, individual innovation factors are not required to increase urban green innovation efficiency. But cities with good openness can attract creative forces and foster open innovation, which is essential for producing high levels of green innovation efficiency in cities. The innovation subject-balanced development model, the innovation environment-innovation asset dual drive model, and the innovation subject-open drive model have all been identified as additional models to support urban innovation efficiency. Finally, we discovered that it is not possible to increase the efficiency of green innovation in the city when each innovation factor in the city is performing poorly, and when there is also a lack of innovation subject and system openness. This study attempts to offer fresh theoretical angles and a variety of urban low-carbon development pathways.

## Introduction

The adoption of the reform and opening-up strategy has accelerated China’s urbanization. Between 1978 and 2021, the number of people living in cities and towns went from 172 to 914 million, and the urbanization rate rose from 17.9 to 64.7% (National Bureau of Statistics of China 2022). The coordinated growth of the urban and rural economies, the closing of the income gap between urban and rural areas, the upgrading of residents’ consumption structures, and the constant emergence of new economic growth points are all benefits of increasing urbanization^1^, which is a key factor in boosting domestic demand. However, as a result of urbanization and industrialization, population growth, the expansion of urban land, and intense socioeconomic activity, there has been an increase in carbon dioxide emissions^2,3^, environmental pollution, and energy demand^4,5^. In order to deal with global climate issues, reduce environmental pollution, and pay attention to the sustainability of economic development^6^, China is actively building a green and low-carbon circular development system, implementing green energy-saving renovation projects in cities and towns, and promoting green and innovative development of cities. Many studies have shown that sustainable development is the focus of all countries in the world. In sustainable development, green innovation is the key to promote the green transformation of the mode of production^7^, adjusting the industrial layout and promote economic growth^8,9^. In contrast to the conventional innovation idea, green innovation places more emphasis on changing industrial processes to ones that use less energy, are more efficient, and recycle waste, which has the twin qualities of having both ecological and socioeconomic benefits^10^. Green innovation in cities efficiency is a key indicator of a city’s green innovation because it incorporates environmental benefits into the process of innovation inputs and outputs with the least amount of energy use and environmental cost to produce the best output^11,12^. Promoting sustainable urban development can be accomplished by increasing the effectiveness of urban green innovation.

These relationships might be complementary or alternative. Although there isn’t a single definition of the innovation ecosystem in academic circles, the innovation ecosystem built using innovation theory and ecosystem thinking can better analyze the intricate urban innovation activities and processes and assist in understanding the inner workings and future course of improvement of urban green innovation efficiency. It is challenging to use conventional approaches to examine the complicated causality and various driving mechanisms that affect how effective the urban green innovation ecosystem is. In order to address these issues, this paper opts for fuzzy set qualitative comparative analysis (fsQCA) and necessity analysis (NCA)^13^. It then examines whether and to what extent various innovation elements are required in order to promote urban green innovation efficiency as well as the most efficient strategy and intricate mechanism for doing so. The rest of the paper is organized as follows: The second part is a literature review, which introduces the research on innovation ecosystems and urban innovation ecosystems. The framework for building an urban innovation ecosystem is included in the third section. The research methods, data sources, and data measurement and calibration are included in the fourth section. The examination of the findings makes up the fifth section. The conclusion, suggested countermeasures, and perspective is included in the sixth section.

## Literature review

### Green innovation efficiency measurement and influencing factors

Understanding the influence mechanism and evolution law of green innovation efficiency will assist support the high-quality growth of the regional economy. Green innovation efficiency is an important indicator to measure the amount of green development in a region^14^. The study of green innovation efficiency now concentrates on two areas: measurement techniques and analysis of affecting factors. On the one hand, there are two primary approaches for measuring the effectiveness of green innovation: the data envelopment analysis approach (DEA) and the stochastic frontier approach (SFA)^15^. SFA is a stochastic boundary model with compound perturbation terms that was developed using a deterministic production function. It has some drawbacks, including strong subjectivity, the inability to perform multiple input and multiple output efficiency values^16^, and the requirement that the user choose the precise form of the production function in advance. The DEA model is a nonparametric approach that encloses the optimal production frontier surface using linear programming and convex analysis theory to examine the relative efficacy of various factors. DEA can somewhat lessen the errors brought on by subjective decisions because it does not assume the functional shape of the optimal production frontier^17^. However, traditional DEA models (CCR and BCC models) do not account for elemental slack, such as excessive unit inputs and insufficient efferent, because they presume that inputs and outputs fluctuate proportionally. In light of this, Tone^18^ presented the SBM model, which adds non-radial slack variables to the objective function and is able to quantify the effectiveness of each decision unit more accurately. On the basis of this, Cooper et al.^19^ built the DEA-SBM model with non-expected outputs, making the input–output index more plausible.

On the other side, scholars have examined how environmental regulation, high-speed railway construction, the Internet, and technological innovation affect the efficiency of green innovation. For instance, the “Porter hypothesis” posits that environmental regulations may initially raise costs for businesses, but ultimately lead to cost reductions and improved product quality, resulting in a competitive advantage and enhanced green innovation capacity. High-speed trains are a major component in fostering innovation growth and have a substantial impact on the development of green innovation efficiency, according to Huang and Wang^20^ analysis of the innovation factor flow perspective. Zhang et al.^21^ used the SBM model to examine how environmental regulation affected the effectiveness of green innovation in the city of Xi’an. They discovered a non-linear inverse U relationship between environmental regulation and the effectiveness of green innovation and that market-based voluntary regulation is better at fostering green innovation. Miao et al.^22^ found that there is a fluctuating growth trend in achievement transformation, technological development, and green innovation in various regions of China, with the effectiveness of green innovation in the eastern region having been in the forefront. According to Wang et al.^23^ research from 2022, the Internet facilitates the gathering of producer services, fosters the growth of the financial sector and is less resource-reliant, and contributes significantly to increasing the effectiveness of urban green innovation. The impact of renewable energy technological innovation (RETI) on the green innovation efficiency of mineral resources is significant, according to Feng et al.^24^, and it varies depending on the industrial structure and level of human capital.

### Urban innovation ecosystem

Innovation ecosystem originates from innovation system^25^. Urban Innovation Ecosystem: An interactive network formed by various departments and institutions within a specific geographical area, emphasizing the dynamic evolution of interactions among innovation actors in complex systems. The concept of an “innovation ecosystem” was first introduced by the President’s Council of Science and Technology Advisors (PCAST) in 2004. The leadership role relies on the dynamic and evolving nature of the “innovation ecosystem”^26^. This concept has garnered significant attention and scholarly investigation. Subsequently, researchers delved deeper into defining and characterizing the innovation ecosystem. Adner^27^ defines an innovation ecosystem as “a collaborative arrangement in which companies combine their respective products into a coherent, customer-oriented solution”. Building upon existing studies, Granstrand^28^ defines an innovation ecosystem as a collection of evolving actors, activities, products, institutions, and relationships. These relationships encompass both complementary and substitution relationships, which play a vital role in the innovation performance of individual actors or a group of actors. The leading position of countries and cities in science, technology, and innovation is contingent upon a dynamic innovation ecosystem. As a result, research on urban innovation has transitioned from focusing on urban innovation systems and innovation cities to the urban innovation ecosystem. Scholars have made varying degrees of progress in researching the urban innovation ecosystem, encompassing concept definition, evaluation and measurement of the current situation, and mechanisms for promoting value. The urban innovation ecosystem refers to a complex ecosystem within a city, where numerous factors interact and depend on each other to collectively foster innovation. The urban innovation ecosystem is a complex network^29^ that encompasses collaboration, integration, and innovation among various stakeholders, such as government entities, businesses, universities, research institutions, investment organizations, and entrepreneurs. The urban innovation system can be seen as a vector set comprising the diffusion effects of urban innovation and the agglomeration effects of the science and technology industry^30,31^, and the process of urban high-quality development is the formation process of urban innovation ecosystem^32^. With regard to the measurement of urban ecosystem, Liu^33^ uses niche evaluation model to construct suitability index to evaluate the development of urban innovation ecosystem in various regions of China. Wolfram^34^ constructed a complex system framework, which contains ten interdependent factors involved in urban development. He uses this framework to empirically evaluate the urban transformation capacity of three cities in South Korea, and puts forward four mechanisms to promote urban sustainable development. Liu^35^ uses BP neural network and improved NK algorithm to determine the optimal evolution path of Beijing innovation ecosystem through simulation. Webb^36^ emphasized the importance of urban innovation ecosystem and proposed a method and framework for jointly formulating national and local strategies to promote the transformation of urban systems.

### Urban Innovation ecosystem and urban green innovation efficiency

There is a complex and interactive relationship between China’s urban innovation ecosystem and green innovation efficiency. Urban innovation ecosystem is a complex system that covers multiple interrelated elements such as innovation subjects, innovation environment, innovation resources, innovation drivers, system openness and innovation The urban innovation ecosystem is a complex system covering several interrelated elements such as innovation agents, innovation environment, innovation resources, innovation drivers, system openness and innovation assets. These elements vary significantly in economic development, policy context and between cities^37^. At the same time, the factors that influence green innovation exhibit diversity and interact with each other, further enhancing the complexity of the system. Therefore, in order to gain a better understanding of the relationship between urban innovation ecosystems and the efficiency of green innovation, it is necessary to explore this relationship while considering the diverse environmental conditions and the complex causal^38^ relationships involved. Initially, researchers have highlighted that the configuration of conditions for achieving high urban innovation capacity is intricate and interconnected. However, there is relatively limited literature that directly depicts the relationship between these conditions. Dong^38^ analyzed the level of green innovation in Chinese cities and its multiple influencing factors by constructing a spatial association network including socio-cultural, policy, and economic factors, and pointed out the role of universities and other innovation agents in promoting green innovation and strengthening spatial association in cities. Huang^39^ studied the innovation capacity of China’s urban innovation system using a triadic subject-resource-environment analysis framework and a fuzzy qualitative comparative analysis method, and found that the mutual matching between various core conditions is the key to improving urban innovation capacity. Secondly, while some research has made advancements in exploring individual elements of innovation within the ecosystem or analyzing the factors that influence the efficiency of green innovation, there still exists a knowledge gap regarding the direct impact of the urban innovation ecosystem on green innovation efficiency. For example, Luo^40^ believes that environmental regulations and foreign direct investment have regional heterogeneity for China’s green innovation. Kuzior^41^, on the other hand, analyzes the national innovation ecosystem parameters in EU member states and Ukraine parameters of the national innovation ecosystem in EU member states and Ukraine, where public financial support and employment impact are considered as one of the key elements to increase the level of sustainable innovation. In addition, Huang^42^ pointed out that social cooperation helps to promote knowledge sharing, resource integration and the formation of common interests by establishing strong social network relationships and strengthening coordination capacity, thus promoting green innovation practices and new product launches, thereby promoting the level of green innovation. Hofman^43^ argues that supply chain collaboration will promote resource sharing, information flow, and collaborative innovation through building trust and cooperation, ultimately driving the realization and development of green innovation; while the government indirectly drives the development of green innovation by providing policy support, resource input, and market incentives to promote the implementation of supply chain collaboration.

### Literature gap

Previous studies have extensively examined the measurement and influencing factors of green innovation efficiency, yet there are still research gaps that need to be addressed. Several shortcomings in previous research can be identified. Firstly, traditional analysis methods often focus on the net effect of individual factors and lack investigation into the impact of multi-factor interactions on urban green innovation efficiency. Secondly, the complexity of the innovation ecosystem, which comprises multiple interconnected elements, makes it challenging to integrate it with traditional analysis methods, resulting in a scarcity of empirical studies on the innovation ecosystem. To address these shortcomings, this study adopts the Qualitative Comparative Analysis (QCA) method to construct an analytical framework for the innovation ecosystem. This framework aims to explore the complex causal relationships and multiple mechanisms of various innovation elements on urban green innovation efficiency. Additionally, as the QCA method can determine the necessity of an individual innovation factor but cannot ascertain the extent to which these factors contribute to green innovation efficiency, we introduce the Necessary Condition Analysis (NCA) method as a complementary and testing condition to supplement and validate the results obtained from the QCA method.

Building upon the aforementioned discussion, this paper aims to contribute new insights in the following areas: (1) In order to address the limitations of existing studies on understanding urban innovation ecosystems, this paper constructs an analytical framework for urban innovation ecosystems. This framework encompasses crucial elements such as innovation subjects, innovation environment, innovation resources, innovation drivers, system openness, and innovation assets. A more comprehensive understanding of the influencing factors and complex mechanisms of urban green innovation efficiency can be achieved by comprehensively considering the interrelationships among these elements. This innovative analytical framework provides a novel theoretical and methodological perspective for urban innovation research. (2) To address the study of complex causal problems, this paper combines the Qualitative Comparative Analysis (QCA), Necessary Condition Analysis (NCA), and Data Envelopment Analysis (DEA) methods. The QCA method is employed to determine the necessity of individual innovation factors, while the NCA method serves as a complementary and testing condition to further validate the robustness and reliability of the research results obtained through the QCA method. Additionally, the DEA-SBM model is applied to measure green innovation efficiency. By integrating these methods, a more comprehensive and accurate assessment of the relationship between urban innovation ecosystems and green innovation efficiency can be achieved, thus providing a more scientific basis for formulating urban innovation policies. (3) Given the significance and representativeness of China’s innovation policy pilot cities, this paper selects these cities as research cases. These cities hold significant status and influence in promoting innovation development, representing a crucial level of China’s innovation capability. Through the study of these cities, a better understanding of the relationship between urban innovation ecosystems and green innovation efficiency can be attained, and valuable references for innovation policy formulation in other similar cities can be provided.

## A pluralistic theoretical framework based on an urban innovation ecosystem

Cities and urban areas are crucial to the process of innovation and entrepreneurship; innovation and entrepreneurship not only occur in cities but are inextricably linked to cities^44^. “Regional” cannot be separated from the innovation system or the innovation ecosystem. The city-based urban innovation ecosystem is becoming a popular issue in innovation theory studies. Scholars have conducted substantial research on the urban innovation ecosystem. To discuss the transformation of European cities into knowledge-based economies, Winden et al.^45^ developed a comprehensive urban analysis framework that includes human capital, industrial structure, quality of life, availability, diversity, scale, and social equity. It has been discovered that urban basic quality and prosperous organizations are crucial for the development of innovative basic knowledge industries. From the perspective of a complex adaptive system, Kroh^46^ exposes the potential mechanisms, innovation barriers, and drivers of urban innovation and development in urban ecosystems. Using the rooted theory, Zhang et al.^47^ discovered that natural, economic, and social factors play a significant role in urban innovation and development. Liu et al.^48^ built an urban innovation ecosystem using the NK algorithm of the BP neural network and the DEMATEL method. This ecosystem has innovative talents, innovative subjects, innovative surroundings, innovative resources, and innovative assets. Due to the urban innovation ecosystem’s complexity, variety, and symbiosis, its theoretical framework is still being studied, and academics have not yet come up with a single framework for analyzing it. So, using the above study results and the real situation in China, this paper builds an urban innovation ecosystem analysis framework, which includes innovation subject, innovation environment, innovation resources, innovation drive, system opening, and innovation assets. Here’s what the exact analysis says:

### Innovation subject

The urban innovation ecosystem is a complex, pluralistic, and coupled system comprised of numerous innovation elements that are interconnected^49^. In the urban innovation ecosystem, there are a variety of heterogeneous innovation subjects, these innovation subjects exist in a particular ecological environment for long-term or short-term interaction and communication, to foster innovation development. It mainly includes the technological innovation subject represented by enterprises, and the knowledge production innovation subject represented by universities, scientific research institutes, and R&D institutions. Knowledge sharing, interactive learning, and the transfer of technology among innovators are crucial innovation-promoting factors^50^. Businesses are the primary source of technological innovation, and any technological advancement will benefit society in terms of products or employment^51^. In the process of urban development, the importance of universities, scientific research institutes, and R&D institutions in creating and disseminating knowledge has grown, and they are regarded as the key to assisting local businesses in engaging in innovation activities^52^.

### Innovation environment

The innovation environment is a vital component of the urban innovation ecosystem, which is embedded in the urban innovation ecosystem and interconnected with various innovation elements to facilitate innovation activities. The innovation environment consists of the business environment, the cultural environment, the market environment, and the regulatory environment, as well as education, public infrastructure, investment, and trade competition, among other conditions^53^. A favorable innovation environment serves as a catalyst for urban innovation activities, has a positive and substantial effect on urban industrial enterprises^54^, and is a crucial factor in ensuring urban sustainable development. Comparing the regional culture and competitive advantages of Silicon Valley and Route 128 led Saxenian^76^ to conclude that the innovation environment has a significant effect on innovation. According to research conducted by Fritsch and Slavtchev^77^, densely populated areas may be conducive to innovative activities if they provide a diversity of interaction opportunities in addition to adequate investment and a wealth of materials and infrastructure.

### Innovation resources

Human resources, technological resources, innovation assets, and so on, are all examples of innovation resources. Innovation resources support innovation subjects in carrying out innovation activities within the urban innovation ecosystem. The circulation of innovation resources among innovation subjects can, on the one hand, foster cooperation and exchanges among innovation subjects, and, on the other hand, assist local businesses in enhancing their competitiveness and innovation capacity^55,56^. Parent and LeSage^57^ discovered that regional innovation growth is dependent not only on human resources devoted to technological research and development, but also on the spatial dependence between innovation-engaged regions. In the urban innovation ecosystem, the interactive and coordinated development of high-tech enterprises, universities, scientific research institutions, and other innovative subjects promotes the effective integration of diverse resources, which is conducive to enhancing the production efficiency of the region and fostering the sustainable growth of businesses^58^.

### Innovation drive

When Schumpeter proposed the innovation theory, he noted that innovation is driven by the pursuit of excess profits and entrepreneurialism, laying the groundwork for future research on innovation power. Urban innovation drive is the driving force and engine of urban economic development^59^. Urban innovation drive can be divided into two aspects: on the one hand, it results from scientific and technological advancement, market competition^60^, and government support^61,62^, among other factors. The internal drivers include entrepreneurship^63,64^, management innovation^65^, and corporate culture^66^. By comparing various types of eco-innovation, Hojnik and Ruzzier^67^ discovered that product eco-innovation, process eco-innovation, organizational eco-innovation, and environmental R&D investments all appear to be driven by the same factors, including regulations, market pull factors, environmental management, and cost savings. Legal and market pressure are the most important factors propelling eco-innovation.

### System opening

The ecosystem for urban creativity is an open system. Under the condition of system openness, innovation subjects within the system can share materials and information with the outside world. This helps the innovation ecosystem move in a higher and more complex way. In particular, the effect of system openness on urban innovation can be broken down into two main parts. On the one hand, system openness helps innovation subjects inside and outside the system establish a synergistic relationship of deep cooperation and mutual benefit, realize cross-regional and cross-level factor flow, improve the efficiency of urban resource integration^68^, and improve the innovation performance of local enterprises^69,70^. On the other hand, opening up the system helps grow the market for innovation factors like talent, science and technology, capital, etc., creating better conditions for urban innovation activities and better services for urban growth Du Chatenier et al.^71^.

### Innovation assets

Innovation assets serve as multipliers for each innovation element in the urban innovation ecosystem, thereby augmenting the ecosystem’s size and development rate^72^. Innovation assets within the urban innovation ecosystem consist of innovation boot camps, innovation industrial parks, university science and technology parks, entrepreneurship bases, business incubators, angel investment, venture capital, and mentorship networks. The presence of innovation assets in a system may increase the number of “collisions” between innovation agents. The collision between various innovation agents will generate new concepts, ideas, and values, which may enhance the innovation agents’ creativity^73^. Consequently, the multiplier effect of innovation assets will increase the collision density of innovation agents and stimulate the exponential development of existing elements of the innovation ecosystem^74^.

**Figure 1:**
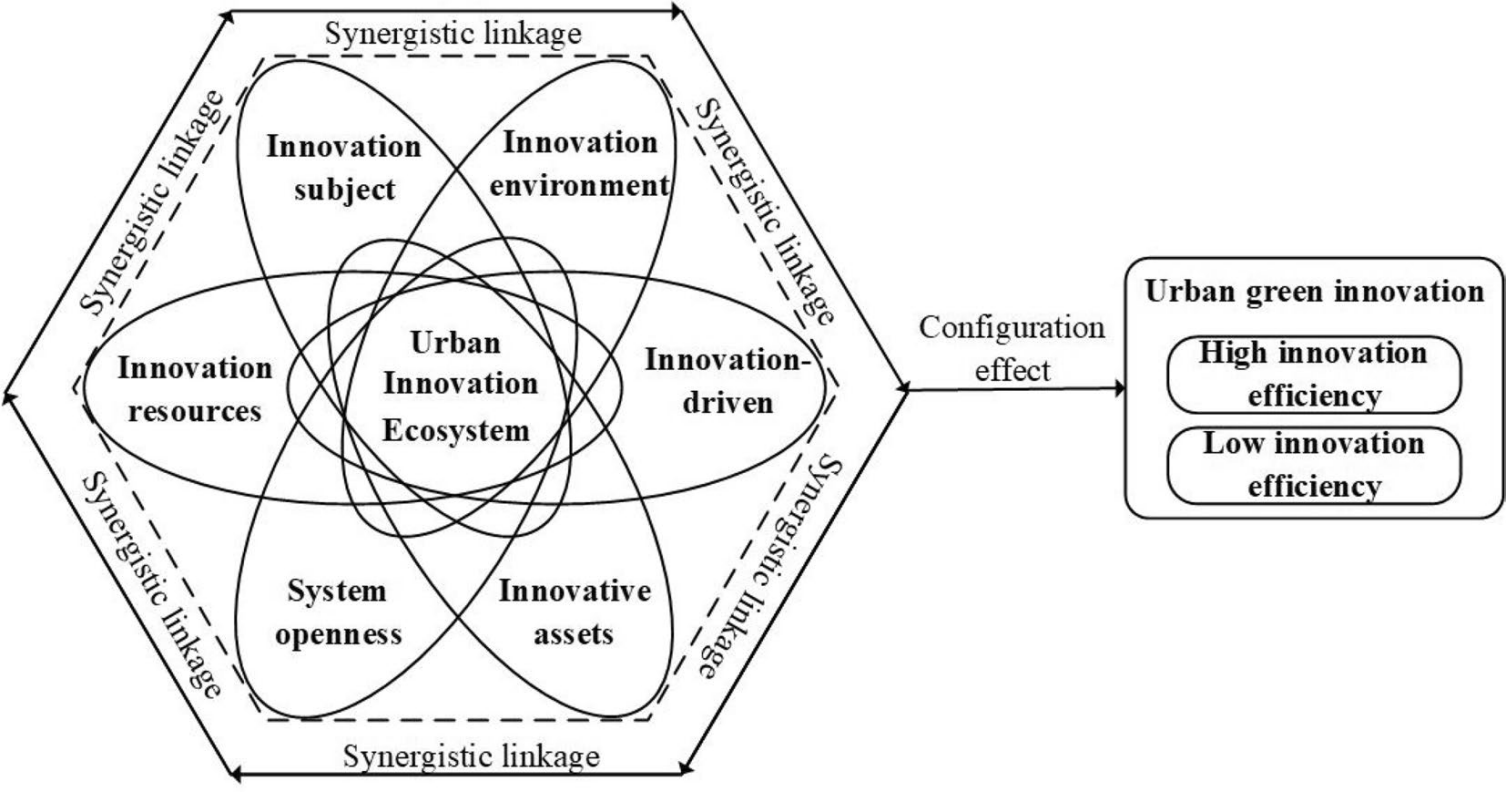
Urban innovation ecosystem drives urban green innovation efficiency mechanism.

In conclusion, this paper considers innovation subject, innovation environment, innovation resources, innovation drive, system openness, and innovation assets as important factors constituting urban innovation ecosystem, and constructs an analysis framework of urban innovation ecosystem containing six conditional variables (see Fig. 1 for a diagram of the framework). Thus, we investigate the multiple driving mechanisms of innovation ecosystem efficacy on urban green innovation.

## Research methods

### DEA-SBM

In this study, the DEA-SBM model is used to calculate the urban green innovation efficiency, including unanticipated output, and the innovative city is used as the decision-making unit to construct the possible urban green innovation efficiency boundary. Suppose there are $h$ decision units in the production system, which include $f$ input variables $x$, ${p}_{1}$ desired outputs $y$, and ${p}_{2}$ undesired output variables $b$. The definition matrix is as follows: $X=\{{x}_{1},...,{x}_{h}\}\in {R}^{f\times h}$, $Y=\{{y}_{1},...,{y}_{h}\}\in {R}^{{P}_{1}\times h}$, $M=\{possibilities\in {R}^{{P}_{2}\times h}$. In period $t$, the set of production possibility set of $h$ decision units are: $P=\left\{(x,y,b)\hspace{0.33em}|\hspace{0.33em}x\ge X\lambda \text{ ,y}\ge \mathrm{Y}\lambda ,b\ge \mathrm{M}\lambda ,\lambda \ge 0\right\}$. Referring to Cooper et al.^75^, the DEA-SBM model with the inclusion of undesired outputs is as follows:

1 $$\begin{aligned} & \theta^{*} = \min \frac{{1 - \frac{1}{f}\mathop \sum \nolimits_{i = 1}^{f} s_{i}^{ - } /x_{in} }}{{1 + \frac{1}{{p_{1} + p_{2} }}\left( {\mathop \sum \nolimits_{r = 1}^{{p_{1} }} s_{r}^{ + } /y_{rn} + \mathop \sum \nolimits_{t = 1}^{{p_{2} }} s_{t}^{a - } /b_{tn} } \right)}} \\ & s.t.\;\left\{ {\begin{array}{*{20}l} {x_{h} = X\lambda + s^{ - } } \hfill \\ {y_{h} = Y\lambda - s^{ + } } \hfill \\ {b_{h} = M\lambda + s^{a - } } \hfill \\ {\lambda ,s^{ - } ,s^{ + } ,s^{a - } \ge 0} \hfill \\ \end{array} } \right. \\ \end{aligned}$$

In Eq. (1), ${\theta }^{*}$ denotes urban green innovation efficiency, $0 < \theta^{*} \le 1$; $s^{ - } ,s^{ + } , s^{a - }$ denote the slack variables for inputs, desired outputs, and non-desired outputs, respectively; $\lambda$ is the weight vector. The larger ${\theta }^{*}$ represents the higher DMU efficiency value, when and only when $s^{ - } = s^{ + } = s^{a - } = 0$ and ${\theta }^{*}=1$ represents strong efficiency effective. When ${\theta }^{*}<1$, it means that the efficiency value is invalid and can be further improved by improving the input and output.

### QCA and NCA

The purpose of this paper is to examine the complex mechanism underlying the relationship between the urban innovation ecosystem and urban green innovation efficiency, and empirical tests will be conducted using fsQCA and NCA. Ragin proposed the QCA in 1987^76^. This method employs Boolean algebra operations to investigate the influence of multiple antecedent configurations on the resulting variables. It is advantageous to describe causal complexity and multiple concurrency mechanisms^77^. Clear set comparative analysis (csQCA), multi-valued set qualitative comparative analysis (mvQCA), and fuzzy set qualitative comparative analysis (fsQCA) are the three fundamental categories of qualitative price comparison analysis. In comparison to csQCA and mvQCA, which are appropriate for dealing with binary variables and more discrete values, fsQCA can analyze the differences in degree changes and set membership, making it appropriate for continuous variable data^13^. Since the innovation ecosystem’s antecedent condition variables and outcome variables are continuous, fsQCA is applicable to this study. However, the QCA method can only determine qualitatively whether the antecedent condition is required for the outcome; it cannot analyze the degree to which the antecedent condition is required for the outcome. In 2016, Dul proposed that the Necessary Conditions Analysis (NCA) could effectively mitigate for the deficiencies of the Quality Conditions Analysis (QCA)^78^. Therefore, this paper will refer to Vis and Dul^79^ to combine NCA and QCA to produce more scientific analysis results.

In this study, the urban innovation ecosystem is comprised of six antecedent variables, including innovation subject, innovation environment, innovation resources, innovation motivation, innovation openness, and innovation activity, with urban green innovation efficiency serving as the dependent variable. First, NCA is used to determine whether and to what extent the urban innovation ecosystem is required to produce urban green innovation efficiency, and the fsQCA method is utilized to determine the robustness of the results of the necessary condition analysis. On this basis, this paper analyzes, from the perspective of configuration, the influence of the combined effect of six antecedent variables of the urban innovation ecosystem on the efficiency of urban green innovation and reveals effective ways for the combination of different elements of the innovation ecosystem to enhance the efficiency of urban green innovation.

### Data source

China has been implementing the pilot policy for innovation-driven cities since 2008 to tap into the potential for urban innovation, explore innovative city models, and enhance urban innovation capabilities. As of March 2022, the Ministry of Science and Technology and the National Development and Reform Commission have supported 103 cities and districts in carrying out innovation-driven city construction. Referring to the definition provided in the “National Innovation-Driven City Innovation Capacity Evaluation Report,” we define innovation-driven cities as those that prioritize scientific and technological innovation as the core driving force for socio-economic development. These cities possess abundant innovation resources, vibrant innovation entities, efficient innovation services, effective government governance, and a favorable environment for innovation and entrepreneurship. They play a significant supportive and leading role in constructing innovative provinces and the nation as a whole. To ensure the scientific, comprehensive, and reliable nature of our research, we selected classic indicators from the “National Innovation-Driven City Innovation Capacity Evaluation Report” and the “China Regional Innovation Capacity Evaluation Report” during the indicator selection process. Considering the feasibility and data quality of data collection, we chose 71 cities out of the 103 innovation-driven cities as our research subjects. For detailed information, please refer to Table 1. The six conditioning variables in the urban innovation ecosystem and the basic data on urban green innovation efficiency were sourced from the “China Statistical Yearbook,” “China Urban Statistical Yearbook,” “China Science and Technology Statistical Yearbook,” the China Research Data Services Platform (CNRDS), and various city statistical yearbooks, as detailed in Table 2.

**Table 1:** List of sample cities.

	City		City		City		City		City
1 2 3 4 5 6 7 8 9 10 11 12 13 14 15	Shijiazhuang Tangshan Qinhuangdao Nanjing Wuxi Xuzhou Changzhou Suzhou Nantong Lianyungang Yancheng Yangzhou Zhenjiang Taizhou Hangzhou	16 17 18 19 20 21 22 23 24 25 26 27 28 29 30	Ningbo Jiaxing Huzhou Shaoxing Jinhua Fuzhou Xiamen Quanzhou Longyan Jinan Qingdao Dongying Yantai Weifang Jining	31 32 33 34 35 36 37 38 39 40 41 42 43 44 45	Guangzhou Shenzhen Foshan Dongguan Haikou Taiyuan Hefei Wuhu Ma’anshan Nanchang Jingdezhen Pingxiang Zhengzhou Luoyang Nanyang	46 47 48 49 50 51 52 53 54 55 56 57 58 59 60	Wuhan Yichang Xiangyang Zhuzhou Hengyang Changsha Hohhot Baotou Nanning Chengdu Guiyang Zunyi Kunming Yuxi Xi’an	61 62 63 64 65 66 67 68 69 70 71	Baoji Hanzhong Lanzhou Xining Yinchuan Urumqi Shenyang Dalian Changchun Jilin Harbin

**Table 2:** Variable description and data sources.

First-level indicator	Second-level indicator	Variable description	Data sources
	Number of R&D personnel among 10,000 employed persons	Measuring the size of urban R&D workforce clusters	National Innovation City Innovation Capacity Evaluation Report
Innovation subject	Number of high and new technology enterprises	Measuring the scale of high-tech enterprises in cities	National Innovation City Innovation Capacity Evaluation Report
	Number of general higher education institutions	Measuring the size of urban research clusters	China City Statistical Yearbook
	Number of technology-based SMEs	Measuring the activity of urban enterprises	National Innovation City Innovation Capacity Evaluation Report
	Total retail sales of social consumer goods	Measuring the level of urban consumer demand	China City Statistical Yearbook
	Natural population growth rate	Measuring the extent and trends of urban population change	China City Statistical Yearbook
	Expenditure on education	Measuring the importance cities place on talent development	China City Statistical Yearbook
	Urban Registered Unemployed Population	Measuring Urban Employment Levels	China Statistical Yearbook
Innovation environment	Added value of the tertiary industry	Measuring the city’s industrial structure	China City Statistical Yearbook
	Year-end cell phone subscriber numbers	Measuring urban communication infrastructure	China City Statistical Yearbook
	Number of Internet broadband access subscribers	Measuring Urban Information Infrastructure	China City Statistical Yearbook
	Road mileage	Measuring urban transportation infrastructure	China City Statistical Yearbook
	Number of college students per 10,000 people	Measure urban human capital	China City Statistical Yearbook
	Number of full-time teachers in colleges and universities	Measuring the extent of urban education resources	China City Statistical Yearbook
Innovation resources	Year-end deposit balance of financial institutions as a percentage of GDP	Measuring the level of urban financial development	China City Statistical Yearbook
	Number of invention patents per 10,000 people	Measuring the City’s Independent Innovation Capability	National Innovation City Innovation Capacity Evaluation Report
	Per Capita Disposable Income of Residents	Measuring market demand	Statistical Yearbook by City
	PM2.5	Measuring the level of urban environment	National Innovation City Innovation Capacity Evaluation Report
Innovation-driven	Amount of science and technology expenditure	Measuring the intensity of government spending on science and technology	China City Statistical Yearbook
	Amount of science and technology expenditure	Measure the income gap between urban and rural areas	Statistical Yearbook by City
	Energy consumption per unit of gross domestic product	Measure the level of urban energy consumption	National Innovation City Innovation Capacity Evaluation Report
	Commercial credit environment index	Measuring the market credit trading environment	China City Business Credit Environment Index
	Actual use of foreign capital per capita	Measuring the level of urban integration	China City Statistical Yearbook
	Number of international Internet users	Measuring the degree of information flow	China City Statistical Yearbook
System openness	Road passenger traffic	Measuring the level of urban passenger transport	China City Statistical Yearbook
	Road freight volume	Measuring the level of city logistics	China City Statistical Yearbook
	Total imports and exports as a percentage of GDP	Measuring a city’s foreign trade dependence	China City Statistical Yearbook
	National-level dual-innovation demonstration base	Measuring the number of innovation assets in a city	Ministry of Science and Technology of the People’s Republic of China Website
Innovative assets	Number of national-level incubatees	Measuring the dynamics of urban innovation transformation	National Innovation City Innovation Capacity Evaluation Report
	Provincial Ministry Co-operative Innovation Center	Measuring the Dynamics of Collaborative Innovation in Cities	Website of the Ministry of Education of the People’s Republic of China

### Measurement and calibration

#### Result variables

This study employs the DEA-SBM model and MaxDEA software to evaluate the green innovation efficiency of 71 innovative cities located in China. The efficiency of urban green innovation can be calculated using two variables: input and output. The input indicators consist of capital stock, labor input, and energy input^23,80^. Capital input is quantified by the capital stock indicator, which is computed through the perpetual inventory method, deflating the total social fixed assets with the fixed asset investment price index, and using 2005 as the reference period. Labor input is measured by taking the average number of employees at the year-end of the previous and current period, following the methodology of Du et al.^81^. The energy input is quantified by the aggregate electricity consumption of the society. The input variables comprise of expected and non-expected output, measured by GDP and the count of green patent applications, respectively. The count of green patent applications is indicative of the urban green innovation capability^82^. The negative output indicator in industrial processes is determined by measuring the emissions of wastewater, sulphur dioxide, nitrogen oxide, and smoke (dust)^83^. These emissions are combined and scored using the entropy value method.

#### Conditional variables

Concerning previous studies, this paper constructs an urban innovation ecosystem with six conditional variables, with the following indicators.

1. Innovation subjects. Measured by four secondary indicators: the number of high-tech enterprises, the number of science and technology-based SMEs, the number of general colleges and universities, and the number of R&D personnel among 10,000 employed persons, reflecting the quantity and quality of innovation subjects in the city.
2. Innovation environment. Measured by eight secondary indicators: total retail sales of social consumer goods, natural population growth rate, education expenditure, number of the urban registered unemployed, the added value of the tertiary industry, number of cell phone subscribers at the end of the year, number of Internet broadband access subscribers, road mileage, etc., and measured from eight aspects: total social consumption, population growth, the importance of education, employment, industrial structure, Internet, urban infrastructure, etc. The city’s innovation environment is measured in eight aspects.
3. Innovation resources. Measured by four secondary indicators: the number of college students per 10,000 people, the number of full-time teachers in general higher education schools, the proportion of year-end deposit balance of financial institutions to GDP, and the number of invention patents per 10,000 people, reflecting the city’s human resources, financial resources, and innovative technology resources.
4. Innovation-driven. Measured by six secondary indicators: per capita disposable income of residents, PM2.5, amount of science and technology expenditure, the ratio of per capita disposable income of urban and rural residents, energy consumption per unit of gross regional product, and business credit environment index, where the ratio of per capita disposable income of residents and disposable income of urban and rural residents reflects the level of market demand driving urban innovation development. PM2.5 and energy consumption per unit of GDP reflect the urban environment and energy use efficiency. For sustainable urban development, Chinese cities have introduced environmental regulatory mechanisms, laws, and regulations to drive energy conservation and emission reduction in enterprises, improve energy use efficiency, and optimize the urban environment from the policy level. The business credit index can measure the degree of credit risk and the potential of city credit economy development, reflecting the city credit market situation. A good credit market environment can promote the development of financial instruments, provide financing support for start-ups and high-tech research, and effectively drive innovation development.
5. System openness. Measured by five secondary indicators, namely the amount of actual foreign capital used per capita, the number of international Internet users, the volume of road passenger traffic, the volume of road freight, and the proportion of total imports and exports to GDP, reflecting the degree of openness and external accessibility of the city.
6. Innovative Assets. Measured by three secondary indicators, namely, the national mass entrepreneurship and innovation demonstration base, the number of national enterprises incubated, and the collaborative innovation center jointly built by provinces and ministries, to reflect the situation of urban innovation assets.

The present study employs the entropy method to measure the weight of the secondary index in the innovation ecosystem. Subsequently, the index values for follow-up computation are established as comprehensive scores of six conditional variables. The entropy method represents an objective approach to assignment, whereby weight coefficients are determined through an assessment of the degree of variation exhibited by each indicator. This methodology serves to effectively circumvent the influence of human factors^84,85^. To standardize the data and facilitate inter-comparison, the technique of polarization normalization is employed to render the aforementioned secondary index data invariant, while simultaneously mitigating the impact of non-positive values. Additionally, the data undergoes panning. The aforementioned equation can be expressed in the following manner:

Calculation method of positive index:

2 $$X_{ij} = \frac{{X_{ij} - \min X_{j} }}{{\max X_{j} - \min X_{j} }} + 0.001$$

Calculation method of negative index:

3 $$X_{ij} = \frac{{\max X_{j} - x_{ij} }}{{\max X_{j} - \min X_{j} }} + 0.001$$

In the above Eqs. (2) and (3): $i = 1,2, \ldots ,m;\,j = 1,2, \ldots ,n_{o} \,\max \,X_{j}$ represent the maximum and minimum values of the $j$th observation, respectively.

Next, calculate the weight and score of entropy.

A. First, build the original indicator data matrix with $m$ cities and $n$ evaluation indicators. The matrix formula is as follows:
  4 $$X = \left\{ {X_{ij} } \right\}_{m \times n} \left( {0 \leqslant i \leqslant m,0 \leqslant j \le n} \right)$$
  In Eq. (4), ${X}_{ij}$ represents the $j$th index value of the $i$th city.
B. The entropy value of the $j$th indicator is:
  5 $$e_{j} = - k\sum\nolimits_{j = 1}^{n} {P_{ij} \ln P_{ij} ,} \quad j = 1,2, \ldots ,n$$
  Among them, ${P}_{ij}={X}_{ij}/{\sum }_{j=1}^{n}{X}_{ij},\hspace{0.33em}\hspace{0.33em}k=1/\mathit{ln}n$, and ${e}_{j}\ge 0$。
C. Calculating the redundancy of information entropy:
  6 $$d_{j} = 1 - e_{j}$$
D. The weights of each indicator were calculated as:
  7 $$w_{j} = \frac{{d_{j} }}{{\mathop \sum \nolimits_{i = 1}^{m} d_{j} }}\quad 0 \leqslant w_{j} \leqslant 1,\quad \sum\nolimits_{i = 1}^{m} {w_{j} = 1}$$
E. Calculate the composite score for each indicator:
  8 $$s_{i} = \sum\nolimits_{j = 1}^{m} {w_{j} \cdot X_{ij} }$$

The weight coefficients of the secondary indicators of innovation subject, innovation environment, innovation resources, innovation drive, system openness and innovation assets calculated by the entropy value method are shown in Table 3.

**Table 3:** Weight of each indicator of innovation ecosystem.

First-level indicator	Second-level indicator	Information entropy value e	Information utility value d	Weight (%)
	Number of R&D personnel among 10,000 employed persons	0.937	0.063	2.368
Innovation subject	Number of high and new technology enterprises	0.839	0.161	6.042
	Number of general higher education institutions	0.885	0.115	4.319
	Number of technology-based SMEs	0.84	0.16	6.012
	Total retail sales of social consumer goods	0.919	0.081	3.054
	Natural population growth rate	0.973	0.027	1.012
	Expenditure on education	0.909	0.091	3.408
	Urban Registered Unemployed Population	0.993	0.007	0.247
Innovation environment	Added value of the tertiary industry	0.905	0.095	3.567
	Year-end cell phone subscriber numbers	0.926	0.074	2.777
	Number of Internet broadband access subscribers	0.938	0.062	2.339
	Road mileage	0.932	0.068	2.569
	Number of college students per 10,000 people	0.904	0.096	3.63
	Number of full-time teachers in colleges and universities	0.876	0.124	4.681
Innovation resources	Year-end deposit balance of financial institutions as a percentage of GDP	0.953	0.047	1.774
	Number of invention patents per 10,000 people	0.908	0.092	3.467
	Per Capita Disposable Income of Residents	0.956	0.044	1.659
	PM2.5	0.97	0.03	1.137
Innovation-driven	Amount of science and technology expenditure	0.806	0.194	7.292
	Ratio of per capita disposable income of urban and rural residents	0.987	0.013	0.48
	Energy consumption per unit of gross domestic product	0.992	0.008	0.319
	Commercial credit environment index	0.973	0.027	1.001
	Actual use of foreign capital per capita	0.9	0.1	3.763
	Number of international Internet users	0.902	0.098	3.669
System openness	Road passenger traffic	0.846	0.154	5.801
	Road freight volume	0.906	0.094	3.534
	Total imports and exports as a percentage of GDP	0.894	0.106	3.974
Innovative assets	National-level dual-innovation demonstration base	0.894	0.106	3.989
	Number of national-level incubatees	0.898	0.102	3.827
	Provincial Ministry Co-operative Innovation Center	0.78	0.22	8.289

#### Variable calibration

This study employs the direct method to calibrate the antecedent condition variables and result variables of the innovation ecosystem to fuzzy sets, as there is a dearth of clear theories and external standards for calibration^86^. Three anchor points are chosen to represent full membership, intersection, and non-membership. The study employs a threshold of 80%, 50%, and 20% for the complete membership point, intersection point, and non-membership point, respectively, based on six condition variables and one result variable^87^. To address the issue of a 0.5 case affiliation resulting in a failure of the affiliation attribution discriminant, this paper proposes adding 0.5 affiliations to 0.001. The calibration information and descriptive statistics for the condition and outcome variables are shown in Table 4.

**Table 4:** Aggregation, calibration, and descriptive analysis.

Set	Fuzzy set calibration			Statistical analysis			
	Completely affiliated	Crossing point	Completely unaffiliated	Mean	Standard layer	Minimum value	Maximum value
Green innovation efficiency	0.555	0.386	0.249	0.457	0.265	0.111	1
Innovation subject	0.051	0.021	0.009	0.0330	0.0318	0.00194	0.158
Innovation environment	0.061	0.038	0.019	0.0435	0.0294	0.00975	0.150
Innovation resources	0.061	0.027	0.012	0.0352	0.0255	0.00202	0.109
Innovation-driven	0.037	0.026	0.018	0.0291	0.0151	0.0117	0.108
System openness	0.052	0.029	0.014	0.0337	0.0220	0.00231	0.0979
Innovative assets	0.066	0.028	0.004	0.0354	0.0342	0.000016	0.134

## Analysis results

### Necessary condition analysis

The NCA method can not only figure out if a certain condition is required for the result, but it can also tell how important that condition is. The amount of effect is used in NCA to judge the necessary conditions to produce specific results. The effect range 13/28 is, with less than 0.1 representing low-level effect, 0.1 to 0.3 representing medium-level effect, and 0.3 to 1 representing high-level effect^78^. In order to judge the necessary conditions using the NCA method, two conditions need to be satisfied: one is that the effect is greater than or equal to 0.1, and the other is that the result of Monte Carlo simulations of permutation tests is significant^88^. The effects of upper-bound regression (CR) and upper-bound envelope analysis (CE) on antecedent condition variables and result variables are investigated in this paper. The analysis results of NCA necessary items can be found in Table 5. According to Table 5, the effects (d) of the innovation subject, innovation environment, innovation resources, innovation drive and innovation assets are all less than 0.1, and the p-value is more than 0.05. The p-value of the system open was less than 0.05, but the effect size was less than 0.1, which did not satisfy the NCA necessity judgment condition. It follows that none of the 6 antecedent conditions of the urban innovation ecosystem is necessary for the efficiency of urban green innovation.

**Table 5:** Necessary condition analysis results of the NCA method.

Condition	Method	Accuracy (%)	Ceiling zone	Scope	Effect size (d)	*p* value
Innovation subject	CR	100	0.000	0.99	0.000	1
	CE	100	0.000	0.99	0.000	1
Innovation Environment	CR	98.6	0.009	0.99	0.009	0.056
	CE	100	0.013	0.99	0.013	0.038
Innovative Resources	CR	95.8	0.008	0.99	0.008	0.034
	CE	100	0.011	0.99	0.011	0.035
Innovation-driven	CR	100	0.000	0.99	0.000	0.343
	CE	100	0.000	0.99	0.000	0.340
System openness	CR	94.4	0.046	1	0.046	0.000
	CE	100	0.050	1	0.050	0.001
Innovative assets	CR	100	0.002	0.97	0.002	0.031
	CE	100	0.004	0.97	0.004	0.023

In Table 6, the results of the study of bottlenecks in the urban innovation ecosystem are looked at in more detail. Through the bottleneck level value, we can figure out a certain level value that hits the maximum observation range of the result and the level value that needs to be met within the maximum observation range of the antecedent condition variable (%)^88^. To reach 70% of the urban green innovation efficiency level, 1.3% innovation environment, 1.2% innovation resources, 7.7% system openness and 0.3% innovation assets are needed, while there is no bottleneck level between innovation subjects and innovation drivers.

**Table 6:** NCA method bottleneck level (%) analysis results (using CR method, NN = unnecessary).

Urban green innovation efficiency	Innovation subject	Innovation Environment	Innovative Resources	Innovation-driven	System openness	Innovatie Assets
0	NN	NN	NN	NN	NN	NN
10	NN	0	NN	NN	NN	NN
20	NN	0.2	NN	NN	NN	NN
30	NN	0.4	NN	NN	NN	NN
40	NN	0.6	NN	NN	0.8	NN
50	NN	0.9	NN	NN	3.1	NN
60	NN	1.1	0.4	NN	5.4	0
70	NN	1.3	1.2	NN	7.7	0.3
80	NN	1.5	2	NN	10	0.5
90	NN	1.7	2.8	NN	12.3	0.8
100	NN	1.9	3.6	1	14.7	1

Table 6 NCA method bottleneck level (%) analysis results (using CR method, NN = unnecessary).

This paper employs fsQCA to examine the essentiality of NCA. The consistency average determines whether the antecedent condition variable is the result variable in fsQCA. A necessary condition of the result variable is identified when the consistency level exceeds the critical value of 0.9 for the antecedent condition variable. The fsQCA3.0 software was utilized to determine the necessity test of a single antecedent condition, with the corresponding results presented in Table 7. The study indicates that the consistency values of individual antecedent variables within the urban innovation ecosystem are below 0.9. The findings are congruent with those obtained through the NCA approach. A singular antecedent variable within the innovation ecosystem is not a prerequisite for enhancing the efficacy of urban green innovation.

**Table 7:** Necessity Test of the single condition of the QCA method.

Conditional variable	High green innovation efficiency		Non-high green innovation efficiency	
	Consistency	Coverage	Consistency	Coverage
Innovation subject	0.762437	0.763932	0.412834	0.407169
~ Innovation subject	0.408329	0.413998	0.760647	0.759139
Innovation environment	0.759922	0.782672	0.415673	0.421416
~ Innovation environment	0.438234	0.432433	0.785633	0.7631
Innovative resources	0.665456	0.683018	0.467348	0.472174
~ Innovative resources	0.485746	0.480908	0.686258	0.668788
Innovation-driven	0.70682	0.723812	0.442646	0.446194
~ Innovation-driven	0.459195	0.45563	0.726008	0.709096
System openness	0.752655	0.770309	0.407439	0.410469
Innovation subject	0.42398	0.420921	0.772004	0.754439
~ Innovation subject	0.659307	0.696487	0.440943	0.458518
Innovation environment	0.487423	0.469701	0.708121	0.671694

### Configuration analysis

This paper employs fsQCA3.0 software to examine the configurations that lead to high efficiency in urban green innovation as well as those that do not. These configurations represent diverse urban innovation ecosystems that attain identical outcomes. This article establishes the original data consistency threshold at 0.8, the PRI consistency threshold at 0.7, and the case frequency at 1, based on the practices of prior scholars^89^. In the middle of the solution, “existing or missing” is the default option due to a lack of conclusive evidence that the antecedent condition affects the result variable. That is, it is assumed that the emergence of a single urban innovation ecosystem can cause urban green innovation efficiency. The nesting relationship between the intermediate solution and the simple solution is compared using software analysis, and the core condition of each solution is identified: the core condition is the coexistence of the simple solution and the intermediate solution, and the edge condition is only in the intermediate solution^90,91^. The results, such as Table 8, reported the configuration results of 6 antecedent conditions. Four conditional configurations lead to the high efficiency of urban green innovation, and each column represents a possible conditional configuration. The overall solution of the four configurations is 0.915, indicating that 91.5% of the urban green innovation efficiency is high. The solution’s overall coverage is 0.603, indicating that the four types of conditional configurations can explain 60.3% of high urban green innovation efficiency cases. Based on the four configurations, we can further analyze the differences between different paths to produce high urban green innovation efficiency in the innovation ecosystem.

**Table 8:** Realizing the configuration of High and non-High Urban Green Innovation efficiency in fsQCA.

Antecedent condition variable	High urban green innovation efficiency				Non-high urban green innovation efficiency	
	H1a	H1b	H2	H3	N1	N2
Innovation subject	●	●	⊕	●	⊗	⊗
Innovation environment	●	●	●	⊕	⊗	⊕
Innovative resources	⊕		⊗	●	⊗	●
Innovation-driven	●	●	⊕	○	⊗	
System openness	●	●	○	●	⊗	⊗
Innovative assets		○	●	⊗	⊕	●
Consistency	0.885	0.937	0.890	0.964	0.844	0.925
Original coverage	0.197	0.488	0.110	0.153	0.420	0.211
Unique coverage	0.029	0.320	0.038	0.0287	0.313	0.104
Overall consistency	0.915	0.864				
Overall coverage	0.603	0.524				

Note: ●represents the presence of a core condition, ⊗ denotes the absence of a core condition, ○ signifies the presence of a marginal condition, and ⊕ indicates the absence of a marginal condition.

#### The urban high green innovation efficiency configuration

Configuration H1a and configuration H1b can be classified into the same category. They constitute the second-order equivalent configuration and have an equivalent substitution effect^90^. High urban green innovation efficiency may be achieved using high innovation subject, high innovation environment, high innovation drive, and high system openness as the core circumstances, and non-high innovation resources as the edge condition, according to Configuration H1a. High urban green innovation efficiency may be achieved using high innovation subject, high innovation environment, high innovation drive, and high system openness as the core conditions, and innovation assets as the edge condition, according to Configuration H1b. When we compare H1a and H1b, we can see that the core conditions are the same, the edge conditions are different, and there is a substitution relationship between the edge conditions. This configuration is known as the innovation subject-balanced development mode. The representative cities of H1a are Dongguan, Foshan, Nantong, and Yantai. To take Dongguan as an example, according to the National Evaluation report on the Innovation capability of innovative cities, Dongguan’s GDP and innovative cities ranked 19th in 2019, with strong innovation-driven (11th), strong transformation ability of innovative achievements (8th), and low original innovation capability (48th). Dongguan also boasts 42 state-level science and technology business incubators, university science and technology parks, mass entrepreneurship and innovation demonstration bases, over 6000 high-tech enterprises, and over 1950 technology-based small and medium-sized enterprises. This demonstrates that Dongguan has a good innovation environment and a driving force for innovation, which can continue to incubate innovative enterprises through innovative assets, promote high-quality urban development, and then improve the efficiency of urban green innovation. The representative cities of configuration H1b are Guangzhou, Shenzhen, Hangzhou and 17 other cities. Take Shenzhen as an example. Shenzhen proposed in its government report in 2012: “Building a dynamic innovation ecosystem.” Then, Shenzhen strives to build a service-oriented government, optimize the business environment, create an innovative and entrepreneurial environment, and stimulate the market’s innovative vitality. High-tech enterprises, as well as small and medium-sized high-tech enterprises, have emerged as the primary drivers of innovation in Shenzhen. According to China’s Xinhua News Agency, 90% of R&D institutions, 90% of R&D personnel, 90% of R&D funds and 90% of invention patents come from enterprises. A good public service and innovation environment, as well as active innovation subjects, have resulted in the emergence of several world-class outstanding enterprises in Shenzhen, such as Huawei, DJI, and Tencent, promoting Shenzhen’s great-leap-forward development in the field of innovation and entrepreneurship. Increase the effectiveness of urban green innovation.

High urban green innovation efficiency can be produced under the core conditions of high innovation environment, high innovation assets, and non-high innovation resources, according to Configuration H2, and high urban green innovation efficiency can be produced under the edge conditions of system opening, non-high innovation subject, and non-high innovation drive. The model is known as innovation environment-innovation asset dual drive mode. Configuration H2 demonstrates that, despite limited innovation resources such as urban R&D personnel and financial capital, we can fully exploit the basic role of the innovation environment and the incubation ability of innovation assets, strengthen urban openness, make use of industrial cooperation and mature technology, improve urban innovation ability, overcome the limitation of a lack of innovation subjects and resources, and promote efficiency. The typical representative cities of configuration H2 are Tangshan and Xuzhou. As an example, consider Xuzhou. The central city of the Huaihai Economic Zone is Xuzhou. Xuzhou was named one of the 27 happiest cities in China in 2019 and 2020, and the safest city in China from 2019 to 2021. In terms of the innovation environment, Xuzhou is implementing the “National Business Environment Model City 3-years Action Plan” and working to improve it. Xuzhou attracts investment and builds high-quality innovation assets on the basis of a good innovation environment. According to the report on the Evaluation of the Innovation capability of National innovative cities, Xuzhou City has built 55 innovation bearing parks and 126 innovative entrepreneurial carriers in 2021, and formulated and issued 115 policies for attracting investment in science and technology. Giving full play to the advantages of its own innovation environment and innovation assets, creating a market environment of fair competition, and opening up the "isolated island" of information is the full flow of innovation factors, driving the coordinated development of the innovation ecosystem and promoting the market-oriented and efficient allocation of production factors. Increase the efficiency of urban green innovation.

High urban green innovation efficiency, according to Configuration H3, can be produced under the core conditions of high innovation subject, high innovation resources, high system openness and non-high innovation assets, and innovation-driven and non-high innovation environment. The mode is known as innovation subject-open drive mode. Configuration H3 demonstrates that, in the absence of innovation assets and a poor innovation environment, cities can improve the innovation ability of innovation subjects, increase innovation output, and improve urban green innovation efficiency by introducing external innovation technology and fostering collaboration between schools and businesses. The typical representative cities of configuration H3 are Changzhou and Dalian. According to the “National Evaluation report on Innovation capability of innovative cities”, Dalian has 1727 high-tech enterprises and 30 ordinary universities in 2019. The number of R&D personnel among ten thousand employees is 113.02, and the number of patent inventions is 21.27. The data presented above demonstrate that Dalian can capitalize on the advantages of innovation resources, highlight the dominant position of enterprise technological innovation, introduce advanced technology to optimize industrial layout, and strengthen joint innovation of enterprises, colleges, and universities, in order to realize the breakthrough innovation drive of the innovation subject and promote the coordinated development of the urban innovation ecosystem.

#### The way to produce the efficiency of urban non-high green innovation

With two histories, Table 8 evaluates the number of histories that create non-high urban green innovation efficiency. First, the grouping N1 demonstrates that when the urban innovation ecosystem’s innovation agents, innovation environment, innovation resources, innovation drivers, system openness, and innovation assets all perform poorly, the urban innovation ecosystem is ineffective, and no high urban green innovation efficiency is generated. Configuration N2 indicates that a city with abundant innovation resources and assets, but lacking in innovation subject and system opening, cannot achieve high urban green innovation efficiency. This study suggests that cities lacking in information and technology infrastructure, as well as high-tech enterprises, are unable to effectively generate urban green innovation, despite having adequate human resources, financial capital, and innovation incubation platforms.

### Green innovation efficiency heterogeneity analysis in eastern, central, and western cities

Regional disparities in green innovation efficiency improvement may arise in Chinese cities located in the eastern, central, and western regions due to variations in economic development, natural resource endowment, and institutional environment. This study categorizes 71 cities into eastern, central, and western regions based on the classification criteria of the National Bureau of Statistics of China. The purpose is to examine the conjecture. The study recalibrated the cases using quartiles as thresholds for fully affiliated, intersection, and fully unaffiliated. The differentiated paths of green innovation efficiency enhancement for cities in different regions were obtained using fsQCA3.0 software, confirming the conjecture. The specific results are presented in Table 9.

**Table 9:** A high-level differential analysis was conducted to evaluate the efficiency of green innovation in Eastern, Central, and Western cities.

Antecedent condition variables	East		Central				West	
	HE1	HE2	HC1	HC2	HC3	HC4	HW1	HW2
Innovation subject	⊗	○	●	●	⊕	●	●	●
Innovation environment		●	●	●	⊕	⊕	●	●
Innovative resources	⊗	●	●	●	⊕	●	●	⊗
Innovation-driven	⊗	●		○	●	●	●	⊕
System openness	●	●	⊗		●	●		●
Innovative assets	●	○	○	○	⊕	⊕	●	⊗
Consistency	0.974	0.863	0.940	0.926	0.951	0.943	0.994	0.98
Original coverage	0.162	0.531	0.303	0.551	0.213	0.135	0.664	0.167
Unique coverage	0.073	0.442	0.120	0.365	0.09	0.012	0.598	0.102
Overall consistency	0.873			0.94			0.991	
Overall coverage	0.605			0.818			0.766	

Note: ● represents the presence of a core condition, ⊗ denotes the absence of a core condition, ○ signifies the presence of a marginal condition, and ⊕ indicates the absence of a marginal condition.

Table 9 displays two pathways for enhancing green innovation efficiency in eastern cities, categorized as HE1 and HE2. HE1 grouping suggests that achieving high efficiency in urban green innovation requires system openness and innovation assets as core conditions, even in the absence of other conditions. When the urban system is open and the innovation assets network is dense, innovation agents, resources, and drivers may not be necessary. The HE2 cluster indicates that a well-rounded approach to innovation, encompassing innovation subjects, environments, resources, drive, openness, and networks, can significantly enhance the efficiency of green innovation in eastern urban areas. The central cities have four driving paths for efficient green innovation, which are referred to as HC1, HC2, HC3, and HC4. Histories HC1 and HC2 focus on innovation as a core driver for the development of green innovation efficiency in urban areas, with particular attention to the innovation subject, environment, and resources. The fundamental conditions of group states HC1 and HC2 are identical, while the boundary conditions differ to attain comparable outcomes. Histogram HC3 is a crucial factor for promoting innovation and system openness, and its presence can enhance the efficiency of green innovation in urban areas, even in the absence of other conditions. Innovation drive and system openness are crucial for enhancing innovation efficiency in central cities, indicating a contrast with eastern cities. Histogram HC4 indicates that the core factors of innovation, including the innovation subject, innovation resources, innovation drive, and system openness, along with the marginal factors of non-high innovation ring and non-high innovation assets, can enhance the efficiency of green innovation in central cities. Two approaches, Histogram HW1 and HW2, can be employed to enhance the efficiency of green innovation in western cities. The results of Histogram HW1 suggest that optimal levels of green innovation efficiency in western cities can be attained by focusing on core conditions such as innovation subject, innovation environment, innovation resources, innovation drive, and innovation assets. The Histogram HW2 indicates that certain conditions, such as innovation subject, innovation environment, system openness, non-high innovation resources and assets, and non-high innovation drive, can enhance the green innovation efficiency of western cities. The above analysis proves that China’s innovation capacity has significant regional heterogeneity, which is the same as that of^92^.

### Robustness test

The robustness of the causal configuration pathways for improving urban green innovation efficiency was examined in this study, with specific information presented in Table 10. Firstly, by adjusting the case frequency threshold from 1 to 2, the resulting four configurations were found to be largely consistent with the current configurations, indicating the reliability of our research findings. Secondly, the PRI (Positive Regime Index) consistency level was adjusted from 0.7 to 0.75, resulting in two configurations that were largely consistent with the existing configurations. This further strengthened our confidence in the research results. Finally, adjustments were made to the calibration anchor points, with complete membership, crossover point, and no membership set at 0.9, 0.5, and 0.1, respectively. As a result, two configurations were obtained that were largely consistent with the existing configurations. The robustness analysis demonstrates the robustness of our analytical results.

**Table 10:** Robustness test.

Antecedent condition variable	High urban green innovation efficiency (Threshold value = 2, PRI = 0.7)				Non-high urban green innovation efficiency (The anchor point is 0.9 0.5 0.1)	
	H1	H2	H3	H4	H1	H2
Innovation subject	●	○	⊗	●	○	●
Innovation environment	●	●	●	⊕	●	⊕
Innovative resources	⊕	○	⊗	●		●
Innovation-driven	●	●	⊕	●	●	○
System openness	●	●	●	●	●	●
Innovative assets	⊗	○	●	⊗	●	⊗
Consistency	0.894	0.944	0.890	0.964	0.951	0.983
Original coverage	0.178	0.471	0.110	0.153	0.548	0.266
Unique coverage	0.033	0.325	0.038	0.028	0.342	0.061
Overall consistency	0.920	0.950				
Overall coverage	0.595	0.609				

Note: ● represents the presence of a core condition, ⊗ denotes the absence of a core condition, ○ signifies the presence of a marginal condition, and ⊕ indicates the absence of a marginal condition.

## Discussion and conclusion

The focus of urban high-quality development research is on how to optimize the innovation ecosystem to improve the efficiency of urban green innovation. Based on previous scholars’ research findings and from the perspective of complex systems, this paper employs DEA, NCA, and QCA methods to investigate the relationship between 71 innovative urban innovation ecosystems and urban green innovation efficiency in China, revealing the multiple paths of urban innovation ecosystems driving urban green innovation efficiency. The main conclusions are as follows: First of all, the urban innovation ecosystem comprises multiple and concurrent conditions that can be categorized into four groups. These groups drive the generation of urban green innovation efficiency and exhibit distinct pathways. The efficiency of urban green innovation is determined by six antecedent conditions, namely: innovation subject, innovation environment, innovation resources, innovation drive, system openness, and innovation assets. It is not essential for a single innovation factor to be present in order to achieve high urban green innovation efficiency. Urban openness promotes the aggregation of innovation elements, facilitates open innovation, and contributes to high green innovation efficiency in cities. Second, three methods exist for enhancing urban green innovation efficiency: the innovation subject-balanced development model, the innovation environment-innovation asset dual drive model, and the innovation subject-open drive model. The subject-balanced driving model highlights the significance of a balanced urban innovation ecosystem in enhancing the efficacy of urban green innovation. The dual driving model of innovation environment and innovation assets suggests that when innovation subjects and resources are lacking, we can enhance the innovation ecosystem by optimizing the innovation environment, fostering collaboration, and emphasizing the incubation of innovation assets. The subject-open drive model for innovation effectively utilizes innovation resources and subject expertise to enhance the efficiency of urban green innovation. This is achieved through innovation-driven policies, increased collaboration, and the integration of external innovation technology. These three patterns explain multiple paths for urban green innovation efficiency improvement, which can be adjusted and selected according to the actual situation and resource conditions of cities, providing flexibility and sustainability for urban green innovation development. They help government managers identify the strengths and weaknesses of urban innovation ecosystem development and develop more targeted policy solutions against the elements in the ecosystem. Each region has unique environmental and resource conditions. By understanding and applying these three models, policy makers can choose the appropriate path according to city characteristics to achieve synergistic economic, social and environmental development and promote the goal of sustainable urban development. Finally, there are clear distinctions between the driving forces behind urban green innovation efficiency in eastern, central, and western cities. In eastern and western cities, there are two tracks, whereas there are four paths in the middle region. In order to increase the effectiveness of urban green innovation, eastern cities place a greater emphasis on system opening and innovation assets, central cities clearly place a greater emphasis on innovation driving and system opening, and western cities place a greater emphasis on innovation subjects, innovation environment, and system opening. This understanding helps us reveal the fundamental reasons for the disparities in innovation ecosystems and socioeconomic development among different cities. It provides crucial insights into the development gaps between cities, highlighting the weak aspects and key challenges within their respective innovation ecosystems. Moreover, it offers valuable insights for cities in the eastern, central, and western regions of China, guiding them in paving their distinctive paths towards innovation-driven development. By comprehensively grasping these disparities, cities can effectively address their specific needs and capitalize on their inherent strengths to establish cities that are efficient, inclusive, sustainable, and eco-friendly^93^. Such understanding also guides the formulation of targeted policies and measures to foster an environment conducive to innovation. Ultimately, it helps cities narrow the development gaps, cultivate robust innovation ecosystems, and propel sustainable socioeconomic progress.

## Suggestions for countermeasures and prognosis for future research

### Countermeasure and suggestion

Reducing environmental pollution and emphasizing the sustainability of economic development have significant positive impacts on long-term economic growth^6^. In the current context of rapid urbanization, urban innovation has become a key driver of economic growth and sustainable development. A well-developed and robust urban innovation ecosystem is a crucial element for urban innovation and transformation, with a vital influence on enhancing a city’s green innovation efficiency. To better leverage the role of the urban innovation ecosystem and promote urban sustainable development, the following are specific policy recommendations outlined in this article:

1. This paper reveals that a single innovation factor is insufficient to drive the improvement of urban innovation efficiency. Different cities, due to varying innovation factors, resource endowments, business environments, and policy support, exhibit diverse paths in enhancing their green innovation efficiency. City policymakers should leverage their unique conditions, exploit the advantages of innovation factor combinations, and stimulate the vitality of the innovation ecosystem to promote efficiency enhancement. For cities like Shenzhen, Guangzhou, and Hangzhou, which have well-developed infrastructure, abundant innovation factors, and mature financial markets, further optimizing the innovation ecosystem, stimulating the vitality of innovation entities, mobilizing the capital market, and enhancing the market’s core role in factor allocation can elevate the overall innovation capacity of the city. In cities with limited innovation factors, it is crucial to maximize the energy efficiency of core innovation elements and promote the development of key elements within the innovation ecosystem using the available resources. For instance, Dalian, with its strong innovation entities and resources, can encourage high-tech enterprises, small and medium-sized technology companies, and universities to unleash their innovative potential, strengthen the commercialization of innovation outcomes, and implement innovation-driven policies. These measures will promote high-quality urban development and enhance urban innovation efficiency.
2. Given the simultaneous occurrence of various conditions in the urban innovation ecosystem, the government should first develop comprehensive innovation policies that emphasize the coordinated development of innovation subjects, innovation environment, innovation resources, innovation drivers, system openness, and innovation assets, and establish a robust innovation environment service system. Secondly, the government can provide financial support and policy guidance, establish platforms and networks for sharing innovation resources, promote the aggregation and mobility of innovation elements, facilitate the convergence and optimal allocation of innovation resources, strengthen collaboration with diverse innovation subjects, and encourage the transfer and transformation of knowledge and technology. Additionally, considering the presence of system openness across the four pathways and its crucial role in enhancing urban green innovation efficiency, urban policymakers should particularly focus on the impact of urban openness on sustainable urban development. By fostering system openness, collaboration and cooperation among multiple regions, industries, and innovation subjects can be promoted, thereby stimulating open innovation. Upholding urban openness, advancing international exchanges and cooperation, establishing open innovation platforms, attracting global innovative talents, and continuously enhancing international openness levels are crucial. Finally, due to the dynamic, complex, and cyclical characteristics of the urban innovation ecosystem, different features will emerge at different stages of urban development. The government should regularly monitor and evaluate the urban innovation ecosystem, promptly identify the current development level and future trends, identify weaknesses and critical issues during urban development, promote the synergy of various elements, and assist cities in finding suitable paths for innovative development, ultimately realizing sustainable development goals.
3. Based on the identified differences in the driving paths for green innovation efficiency in Eastern, Central, and Western regions, the country needs to adopt a global perspective in policy formulation to enhance communication and cooperation among different regions, promoting the flow and sharing of innovation factors. Establishing cross-regional innovation networks and platforms is essential, encouraging collaboration between cities in the Eastern region and those in the Central and Western regions to jointly drive technological innovation and green development. Furthermore, specific policy measures should be tailored to the characteristics of each region. For the Eastern region, the focus should be on improving system openness and the utilization efficiency of innovation assets, attracting and aggregating innovation resources, and fostering innovative entities. For the Central region, emphasis should be placed on innovation drive and system openness, deepening industry-academia-research collaboration, and enhancing the flow capacity of urban innovation resources. Regular events such as cooperation negotiations for cutting-edge research projects and technology forums should be held to strengthen the talent mobility mechanism among different entities, including the government, academia, research institutions, and enterprises, facilitating the effective flow and sharing of knowledge, technology, and funding. Ren (2018)^94^ argues that the shortage of high-quality talent is a significant factor contributing to the low urbanization efficiency and hindering the sustainable development of cities in southwestern China. The Western region urgently requires the proactive absorption of scientific and technological resources and innovative talents to activate innovation vitality. Under the new round of revitalization strategy, efforts should be made to address the issue of "institutional path dependence," further enhancing the talent development environment and technology transfer capabilities, and attracting external innovative technologies and resources.

### Deficiency and prospect

This paper has some shortcomings that require further improvement in the future. This paper examines 71 innovative cities in China as a case study. However, the conclusion may lack universality and breadth. In future research, the scope of the case may be broadened to encompass additional cities from various countries and regions. This paper limits the antecedents of the innovation ecosystem to six factors due to the complexity and accessibility of data. These factors include innovation subject, innovation environment, innovation resources, innovation drive, system openness, and innovation assets. Innovation elements may be omitted and subsequently integrated into other elements. This paper solely examines the static correlation between the innovation ecosystem and the efficiency of urban green innovation. Researchers can analyze the dynamic relationship between the innovation ecosystem and urban innovation efficiency, as well as the variation in catch-up speed of innovation efficiency among different cities.

### Supplementary Information

Supplementary Information.

## Data Availability

All data generated or analyzed during this study are included in this published article [and its supplementary information files].
