# Author Correction: Green innovation efficiency and multiple paths of urban sustainable development in China: multi-configuration analysis based on urban innovation ecosystem

**DOI:** 10.1038/s41598-023-41736-8

**Published:** 2023-09-05

**Authors:** Jinguang Guo, Yu Fu, Xuefu Sun

**Affiliations:** 1 School of Public Administration, Dongbei University of Finance and Economics, No. 217, Jianshan Street, Shahekou District, Dalian, 116025 Liaoning China; 2 School of Public Finance and Administration, Harbin University of Commerce, No.1, Xuehai Street, Songbei District, Harbin, 150000 Heilongjiang China

Correction to: *Scientific Reports* 10.1038/s41598-023-40084-x, published online 10 August 2023

The original version of this Article contained an error in Affiliation 1, which was incorrectly given as ‘Institute of Public Management, Dongbei University of Finance and Economics, No. 217, Jianshan Street, Shahekou District, Dalian, 116025, Liaoning, China’. The correct affiliation is listed below:

School of Public Administration, Dongbei University of Finance and Economics, No. 217, Jianshan Street, Shahekou District, Dalian, 116025, Liaoning, China.

Additionally, Affiliation 2 was incorrectly given as ‘Institute of Public Finance and Administration, Harbin University of Commerce, No.1, Xuehai Street, Songbei District, Harbin, 150000, Heilongjiang, China’. The correct affiliation is:

School of Public Finance and Administration, Harbin University of Commerce, No.1, Xuehai Street, Songbei District, Harbin, 150000, Heilongjiang, China.

Furthermore, the original version of this Article contained errors in the Acknowledgements section.

“This study was funded by the National Natural Science Foundation of China under the project “Mechanistic Analysis and Policy System Design of the Complex Architecture of Intergenerational Intervention for Poverty of Young Innovative Talents” (71774027); the National Social Science Foundation of China under the project of “Research on the Long-term Mechanism of Relative Poverty Alleviation” (20&ZD169); the Liaoning Project funded by the “Hundred Million Talents Project”; Liaoning Social Science Planning Fund Project “Research on the Mechanism of Dynamic Integration of Multiple Identities in the Dual Transformation and Innovation of State-owned Enterprises in Liaoning Province” (L20AGL010); Liaoning Province Economic and Social Development Research Project “Research on Enhancing Endogenous Dynamics of Rural Revitalization in Liaoning Province” (2022lslybkt-054) was funded by the Liaoning Provincial Social Science Planning Fund. The authors would like to thank the anonymous reviewers for their helpful suggestions and corrections to the first draft of the paper. They have provided useful suggestions and corrections to an earlier draft of our paper.”

now reads:

“This study was funded by the 'National Natural Science Foundation of China' (Grant numbers: [72274029] and [71774027]) and 'The Liaoning BaiQianWan Talents Program’. The authors would like to thank the anonymous reviewers for their helpful suggestions and corrections to the first draft of the paper. They have provided useful suggestions and corrections to an earlier draft of our paper.”

The original Article has been corrected.

